# Di-(2-Ethylhexyl) Phthalate Increases Obesity-Induced Damage to the Male Reproductive System in Mice

**DOI:** 10.1155/2018/1861984

**Published:** 2018-05-17

**Authors:** Jian Zhao, Shi Ren, Chunyu Liu, Li Huo, Zheng Liu, Lingling Zhai

**Affiliations:** ^1^Department of Pharmacology, Shenyang Pharmaceutical University, No. 103 Wenhua Road, Shenhe District, Shenyang, Liaoning 110016, China; ^2^Department of Nutrition and Food Hygiene, Liaoning Center for Disease Prevention and Control, Shenyang, Liaoning 110001, China; ^3^Department of Maternal and Child Health, School of Public Health, China Medical University, Shenyang, Liaoning 110001, China

## Abstract

**Objective:**

This study evaluated the effects of di-(2-ethylhexyl) phthalate (DEHP) and obesity on male reproductive organ function in male mice and the potential mechanism of male secondary hypogonadism (SH) in such mice.

**Methods:**

140 mice were assigned to six groups for 12 weeks: normal, DEHP, DIO, DIO + DEHP low, DIO + DEHP middle, and DIO + DEHP high. The effects of DEHP and obesity upon the reproductive organs were determined by measuring sperm count and motility, relative testis and epididymis weight, hormone level, and pathological changes. Oxidative stress was evaluated by determining malondialdehyde, T-AOC, SOD, GSH, H_2_O_2_, CAT, and GSH-PX in testicular tissues. Nrf2 and Keap1 protein were measured by Western blotting.

**Results:**

DEHP and obesity reduced sperm count and motility, relative testis and epididymis weight, and testosterone level but increased the levels of MDA, H_2_O_2_, leptin, and estradiol. Pathological injury was observed in the testicular Leydig cells. Moreover, the activity of CAT, SOD, and GSH-Px enzymes was inhibited. Nrf2 protein expression was reduced but that of Keap1 was increased.

**Conclusions:**

DEHP and obesity jointly caused damage to male productive function. Oxidative stress in testicular tissue, and a high level of leptin, may provide some evidence to clarify the mechanisms of male SH with DEHP and obesity.

## 1. Introduction

Obesity is a multifactorial condition with syndromic and nonsyndromic variants. During 2011–2014, the prevalence of obesity in adults in the United States of America was over 36% [1]. In contrast, a 2014 study of chronic disease and nutrition in the Chinese population revealed that the prevalence of obesity and excessive weight gain was 11.9% and 30.1% among adults [2]. Moreover, the prevalence of obesity and excessive weight gain in adults had increased by 230% and 84% since 1992, respectively; further increases in obesity are expected in the future [2].

Previous studies have shown that obesity has an impact upon male reproduction [3]. For example, male obesity is associated with an increased incidence of low sperm concentration and a progressively low motile sperm count [3]. Even in the absence of organic disease in the hypothalamo-pituitary axis, the prevalence of secondary (hypogonadotropic) hypogonadism (SH) in obese men has also been demonstrated in several studies [4–6]. The pathogenesis and clinicopathological correlates of obesity-associated SH have not been fully elucidated yet. The mechanisms involved in the association of male SH and obesity are complex. However, male obesity has been associated with lower plasma testosterone levels [7, 8]. Since the development of the male reproductive organs and male secondary sexual characteristics is promoted by androgens, and since spermatogenesis is closely related to androgen secretion, it follows that reduced levels of testosterone may contribute to male SH in obesity [9].

Endocrine disrupting chemicals are exogenous substances that have the ability to change endocrine function and cause adverse effects at the level of the organism, its progeny, and/or (sub) populations of organisms; these chemicals can cause the abnormal development of reproductive organs and reproductive dysfunction [10]. Di-(2-ethylhexyl) phthalate (DEHP), a form of endocrine disrupting chemicals, is widely used as a plastic plasticizer for synthetic polymers. Humans are widely exposed to DEHP, because of its use in many daily products, including vinyl flooring, wall covering, plastic bags and covers, food containers, cosmetics, and toys [11]. Therefore, obese people can easily come into contact with DEHP. Worryingly, DEHP has well-documented antiandrogenic effects [11]. In China, it is common for obese men to be exposed to DEHP, and we should therefore consider the effects of such exposure on androgens. We hypothesized that there is likely to be a joint action between obesity and DEHP upon male reproduction and that low levels of testosterone levels might be the key mechanism underlying this effect.

Leptin is considered to be the most important factor in regulating the reproductive axis, and high leptin levels have been found in obese males [12]. Our previous study found that high leptin level was one of the mechanisms responsible for reducing the level of testosterone in obese males [9]. However, it remains unclear as to what the exact changes are in obese males exposed to DEHP. We therefore wanted to investigate whether leptin levels are the key factor regulating testosterone levels in obese males exposed to DEHP.

Oxidative stress has also been found to be a highly influential factor upon male reproduction [13]. In our previous study, we proved that oxidative stress can damage testicular tissue in obese males. Therefore, in this present study, we attempted to ascertain the effect of obesity and DEHP on the function and development of reproductive organs in male mice. In addition, we evaluated the possible mechanisms (high leptin level and oxidative stress in testicular tissue) underlying the joint-damaging effect of obesity and DEHP upon the male reproductive system.

## 2. Materials and Methods

### 2.1. Animals, Diet, DEHP Exposure, and Grouping

#### 2.1.1. Animals

A total of 140 4/5-week-old C57BL/6J male mice were obtained from the Experimental Animal Center, China Medical University, Shenyang, China. Mice were fed standard laboratory chow for the 1st week to allow them to adjust to their new environment. Animals were housed individually in a temperature and humidity-controlled room (25 ± 2°C and 55 ± 10%, resp.) on a 12-hour light/dark cycle with free access to food and water. All experimental procedures were conducted in conformity with the institutional guidelines for the care and use of laboratory animals in China Medical University, Shenyang, China, and conformed to the National Institutes of Health Guide for Care and Use of Laboratory Animals (publication number 85-23, revised 1985). All efforts were made to minimize the number of animals used and their suffering.

#### 2.1.2. Diet

Mice were randomly assigned to a standard laboratory diet (10% calories from fat, 20% calories from protein, and 70% calories from carbohydrates, 3.85 kcal/g) (*n* = 20) or a home-made high-fat diet, which contained 45% kcal from fat, as the high-fat diet group (*n* = 120) [9]. The high-fat diet was made up of 73% standard chow diet plus 20% lard, 7% casein (Aoboxing Biotech Company Ltd., Beijing, China) and trace amounts of multiple vitamins.

#### 2.1.3. DIO (Diet-Induced Obesity) Definition

After 8 weeks on high-fat diets, the 40 mice in the upper tertile of body weight gain (which had been fed the high-fat diet) were defined as DIO mice, according to the method used by Levin et al. [14]. The remaining 80 mice in the intermediate and lower tertile of body weight gain were discarded from this study.

#### 2.1.4. DEHP Exposure

After 8 weeks of feeding on a normal or high-fat diet, 10 mice fed upon the normal diet were given an oral gavage once a day for 4 weeks with 100 mg/kg of DEHP. 30 mice (*n* = 10 each group) fed the high-fat diet were also given an oral gavage once a day for 4 weeks with different doses of DEHP (30 mg/kg body, 100 mg/kg body, and 300 mg/kg body).

#### 2.1.5. Grouping Methodology

Six different groups of mice were created and analyzed in this study.

1. Ten mice fed a normal diet for 12 weeks were defined as the normal group (normal group).
2. Ten mice fed a normal diet for 12 weeks and exposed to DEHP (100 mg/kg body weight) for 4 weeks (from the 8th week) were defined as the DEHP exposure group (DEHP group).
3. Ten DIO mice exposed to a high-fat diet only for 12 weeks were defined as the DIO group (DIO group).
4. Ten DIO mice exposed to a 12-week high-fat diet and 4 weeks of DEHP (30 mg/kg body weight, from the 8th week) were defined as the high-fat and DEHP low exposure group (DIO + DEHP low group).
5. Ten DIO mice exposed to a 12-week high-fat diet and 4 weeks of DEHP (100 mg/kg body weight, from the 8th week) were defined as the high-fat and DEHP middle exposure group (DIO + DEHP middle group).
6. Ten DIO mice exposed to a 12-week high-fat diet and 4 weeks of DEHP (300 mg/kg body weight, from the 8th week) were defined as the high-fat and DEHP high exposure group (DIO + DEHP high group).

### 2.2. Experimental Procedures

**Figure 1:**
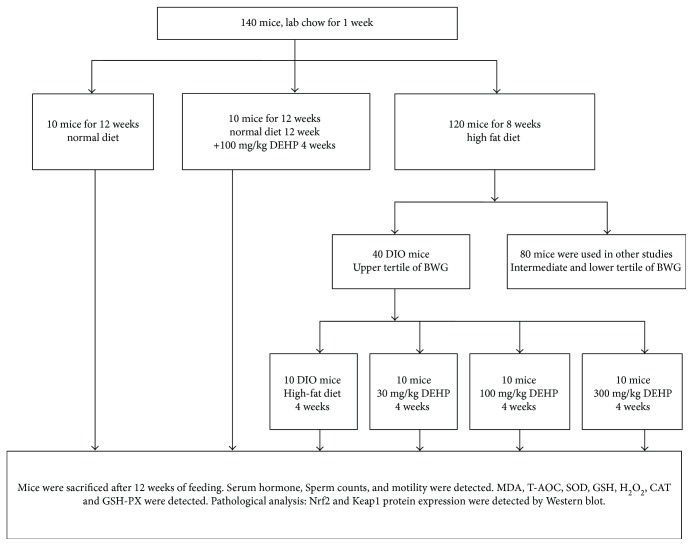
The flowchart of the animal experiment. BWG: body weight gain.

As shown in Figure 1, 140 mice were allowed to adjust to their new environment for 1 week. Ten mice were fed a normal diet for 12 weeks. Ten mice were fed a normal diet for 12 weeks and then exposed to DEHP for 4 weeks. The remaining 120 mice were fed a high-fat diet for 8weeks. Then, 40 mice were defined as DIO mice. Ten of the DIO mice were exposed to a high-fat diet for 4 weeks. The other 30 DIO mice were exposed to both a high-fat diet and DEHP at different doses (30, 100, and 300 mg/kg body weight). All mice were sacrificed after 12 weeks of feeding.

### 2.3. Tissue Processing and Assays

Twenty-four hours after receiving the last dose, animals were anesthetized with ether and blood samples were obtained from the vena cava. Serum was separated from whole blood for the measurement of hormones (testosterone, estradiol, and leptin). Immediately after blood samples were collected, the epididymis was rapidly excised, and sperm count and motility were analyzed. Retroperitoneal fat, epididymal fat, epididymis, testis, kidney, and liver were each dissected and weighed.

One testicle of mice (*n* = 10/group) in each group was prepared for 5% or 10% homogenate in order to determine the MDA, T-AOC, SOD, GSH, H_2_O_2_, CAT, and GSH-Px levels; five testicles from the 10 mice in each group were immediately frozen at −80°C for protein expression studies. Five testicles from the 10 mice in each group were prepared for light microscopy and transmission electron microscopy.

All substance contents and enzyme activities were normalized to the protein which was measured by the method described by Deng et al. [15] using bovine serum albumin as a standard. Each sample was tested in duplicate.

### 2.4. Cauda Epididymal Sperm Count and Motility Measurements

Male C57BL/6J mice (fed 12 weeks) were weighed and anesthetized. The left epididymis was immediately removed. The epididymis and the vas deferens were dissected away from the fat. In a six-well plate, the epididymis and vas deferens from each animal were placed in a well containing 1.0 ml of M2 buffer. The epididymis was then cut at the junction between the corpus and cauda epididymis, and the cauda was placed into a well with 1.0 ml of M2 buffer. Several cuts were made in the cauda epididymis with scissors, and the tissue was gently pressed to release sperm. Sperm was also expressed from the vas deferens in a separate well and then removed from the plate. The pressed sperm from the cauda epididymis was collected in an Eppendorf tube. Using a hemocytometer, sperm counts were determined as the number of sperm per microliter.

Sperm count and motility were assessed in accordance with World Health Organization (WHO) guidelines [16] (≥200 sperm counted for each sample). Sperm count was determined by counting on a hemocytometer. Sperm motility was assessed blinded under a light microscope, classifying 200 sperm per animal as either progressive motile, nonprogressive motile, or immotile. Motility was then expressed as a percentage of the total motile population (progressive motility and nonprogressive motility). Detailed methods can be found in our prior study [9].

### 2.5. Pathological Analysis [9]

#### 2.5.1. Light Microscopy

A portion of each testicle was cut into 4 *μ*m thick pieces and fixed in 4% paraformaldehyde. Regular hematoxylin and eosin (HE) staining was performed for morphological observation with an AX-70 microscope (Olympus, Japan).

#### 2.5.2. Transmission Electron Microscopy

A portion of each testicle was cut into fragments (1 mm × 1 mm × 1 mm), fixed in 2.5% glutaraldehyde made up in 0.1 M phosphate buffer (pH 7.2), postfixed in 1.0% OsO_4_, dehydrated in a progressive ethanol and acetone solution, embedded in Epon812, sectioned with an LKB ultramicrotome, and stained with uranyl acetate followed by lead citrate, then observed by H-600 microscopy and photographed.

### 2.6. Hormone Detection

Leptin, testosterone, and estrogen were detected by ELISA methods. All methods were performed according to the instructions provided with the ELISA kit. The leptin kit was purchased from Merck Millipore (Parmstadt, Germany), the testosterone kit from Enzo Life Science Inc. (NY, USA), and the estradiol kit from Cayman Chemical Company (Ann Arbor, MI, USA).

### 2.7. MDA, T-AOC, SOD, GSH, H_2_O_2_, CAT and GSH-PX Assays

Analysis kits for MDA, T-AOC, SOD, GSH, H_2_O_2_, CAT, and GSH-PX assays were provided by Beyotime Biotechnology (Jiangsu, China). The GSH-PX, CAT, SOD, H_2_O_2_, and T-AOC contents were measured using assay kits in strict accordance with the manufacturer's instructions. MDA contents were expressed as nmol·mg^−1^ protein, T-AOC, SOD, CAT and GSH-PX contents were expressed as U·mg^−1^ protein, GSH contents were expressed as mgGSH·g^−1^ protein and H_2_O_2_ content was expressed as mmol·g^−1^ protein.

### 2.8. Western Blotting

The testes were washed twice in ice-cold phosphate-buffered saline (PBS). RIPA buffer (50 *μ*L) was supplemented with 1 mmol/L PMSF, 1 *μ*g/mL of leupeptin, 1 mmol/L *β*-glycerophosphate, 2.5 mmol/L sodium pyrophosphate, and 1 mmol/L Na_3_VO_4_ and placed on ice for 20 min, followed by centrifugation for 20 min at 12,000*g* and 4°C. Next, 50 *μ*g of total protein from each sample was resolved on 10% sodium dodecyl sulfate-polyacrylamide gels and transferred to polyvinylidene fluoride membranes. After blocking in PBST containing 4% skimmed milk for 2 h at room temperature, the polyvinylidene fluoride membranes were incubated with rabbit polyclonal anti-Nrf2 antibody (ab31163, diluted 1 : 1000; Abcam) and anti-keap1 antibodies (ab119403, diluted 1 : 1000; Abcam) in PBST overnight at 4°C. The membranes were then washed three times in PBST and incubated in peroxidase-conjugated AffiniPure secondary antibodies (diluted 1 : 5000; ZSGB-BIO, Beijing, China) in PBST for 2 h at room temperature. Detection was carried out by chemiluminescence using ECL solution (Thermo Fisher Scientific, Waltham, MA, USA). Each sample was in triplicate, at least.

## 3. Results

### 3.1. Body Weight

**Figure 2:**
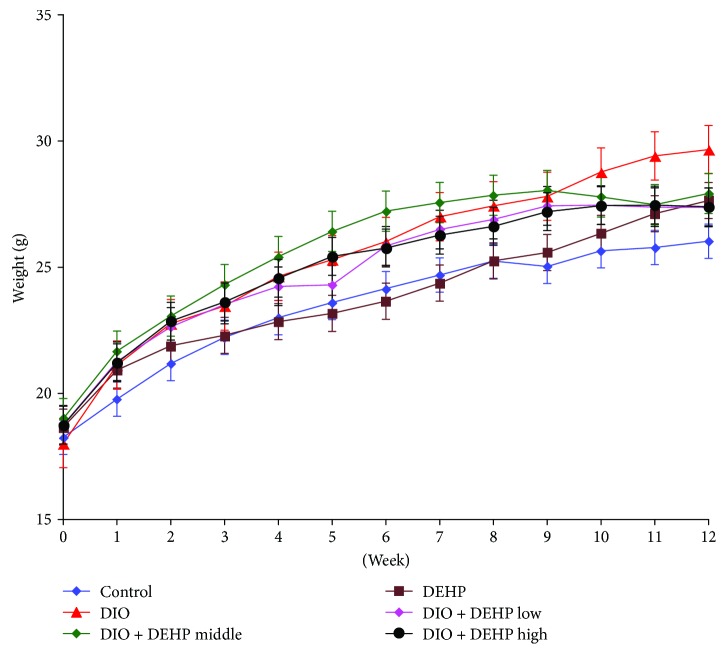
The body weight changes in the six experimental groups in week 12.

A higher body weight was observed in DIO (27.43 ± 1.60 g), DIO + DEHP low (26.89 ± 1.38 g), DIO + DEHP middle (27.85 ± 1.24 g), and DIO + DEHP high (26.62 ± 1.28 g) mice in comparison to age-matched controls (25.24 ± 1.80 g) and DEHP (25.25 ± 0.99 g) mice at 8 weeks (*P* < 0.05). At 12 weeks, the weight of the DEHP (27.64 ± 1.34 g), DIO (29.66 ± 2.39 g), and DIO + DEHP middle (27.92 ± 1.10 g) mice was higher than the control mice (*P* < 0.05). The weight of the DIO mice was significantly higher than the other 5 groups of mice (*P* < 0.05) (Figure 2).

### 3.2. Reproductive Organs, Sperm Count and Motility, and Sex Hormone Levels in the 6 Experimental Groups of Mice

DEHP, DIO, DIO + DEHP low, DIO + DEHP middle, DIO + DEHP high mice and control mice did not exhibit significant differences in the absolute mean weight of testes, epididymis, or seminal vesicles at 12 weeks (not shown in the results). However, as shown in Table 1, there was a significant reduction in the relative testis and epididymis weight in the DEHP, DIO, DIO + DEHP low, DIO + DEHP middle, and DIO + DEHP high mice compared to the control mice (*P* < 0.01). There was also a significant reduction in the relative epididymal weight of the DIO + DEHP middle and DIO + DEHP high mice compared with the DEHP mice (*P* < 0.05). Also, the relative epididymis weight in the DIO + DEHP high mice was lower than that in the DIO, DIO + DEHP low, and DIO + DEHP middle mice (*P* < 0.01).

As shown in Table 1, there was a significant reduction in the relative liver and kidney weight in the DEHP, DIO, DIO + DEHP low, DIO + DEHP middle, and DIO + DEHP high mice compared to the control mice (*P* < 0.01). There was a significant reduction in the relative liver weight of the DIO + DEHP low mice compared with the DEHP mice (*P* < 0.05). Also, the relative liver and kidney weight in the DIO + DEHP middle and DIO + DEHP high mice was lower than that in the DEHP and DIO mice (*P* < 0.05).

As also shown in Table 1, there was a significant increase in the relative epididymal and retroperitoneal fat weight in the DEHP, DIO, DIO + DEHP low, and DIO + DEHP middle mice compared to the control mice (*P* < 0.05). There was a significant decrease in the relative epididymal fat weight in the DIO + DEHP middle and DIO + DEHP high mice compared with the DEHP mice (*P* < 0.05). There was a significant decrease in the relative epididymal and retroperitoneal fat weight in the DIO + DEHP low, DIO + DEHP middle, and DIO + DEHP high mice compared with the DIO mice (*P* < 0.05), and there was a significant decrease in the relative retroperitoneal fat weight in the DIO + DEHP high mice compared with the DEHP mice (*P* < 0.05).

Furthermore, as shown in Table 1, there was a significant decrease in sperm motility and sperm count in the DEHP, DIO, DIO + DEHP low, DIO + DEHP middle, and DIO + DEHP high mice compared to the control mice (*P* < 0.01). There was a significant decrease in sperm motility and sperm count in the DIO + DEHP low, DIO + DEHP middle, and DIO + DEHP high mice compared with the DEHP mice (*P* < 0.05). In addition, sperm motility in the DIO + DEHP low, DIO + DEHP middle, and DIO + DEHP high mice was lower than that in the DIO mice (*P* < 0.05) and the sperm count of the DIO + DEHP high mice was lower than that in the DIO mice (*P* < 0.05).

### 3.3. Light Microscopy in the 6 Experimental Groups of Mice

**Figure 3:**
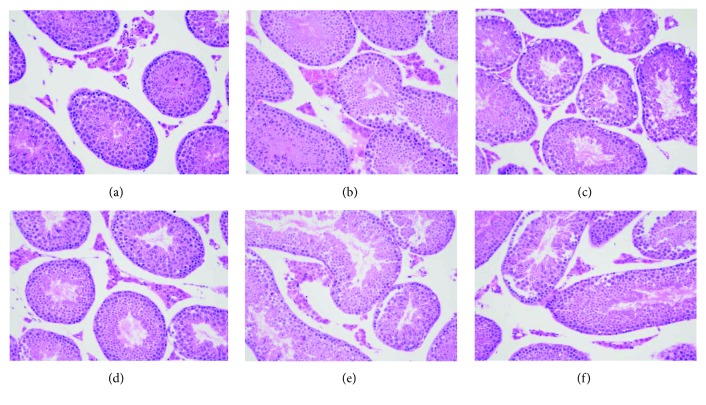
Light microscopic changes of the Leydig cells in six experimental groups in week 12. Light microscopic images showing morphological changes in testicular cells in week 12. Images show the control group (a), DIO group (b), DEHP group (c), DIO + DEHP low group (d), DIO + DEHP middle group, and (e) DIO + DEHP high group (f). Sections were stained with HE staining. Magnification ×40.

To confirm the effects of exposure to the high-fat diet and DEHP on morphological changes in testicular tissue, we performed HE staining. Light microscopic images showed that morphological changes had occurred in the testicular cells after 12 weeks (Figure 3). In the control group (Figure 3(a)), the structure of the seminiferous tubules was normal and complete with slight edema in the Leydig cells. The arrangement of Sertoli cells and germ cells appeared to be slightly irregular in the DIO (Figure 3(b)), DEHP (Figure 3(c)), DIO + DEHP low (Figure 3(d)), and DIO + DEHP middle (Figure 3(e)) groups. In the DIO + DEHP high group (Figure 3(f)), the Leydig cells showed edema. The number and lines of Sertoli cells and germ cells reduced obviously.

### 3.4. Electron Microscopy in the 6 Experimental Groups of Mice

**Figure 4:**
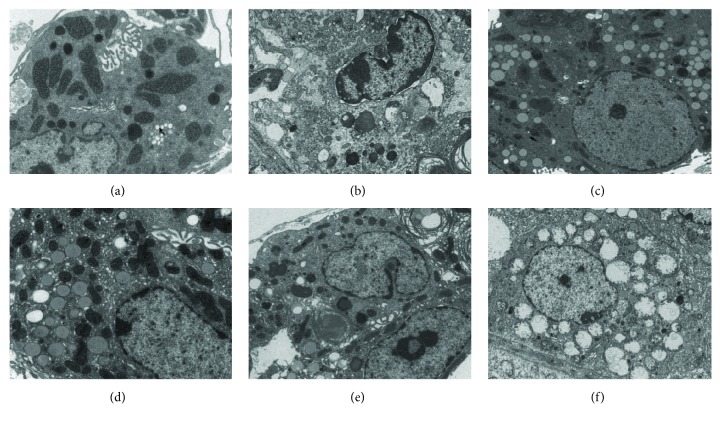
Electron microscopy changes in Leydig cells of the six groups of mice in week 12. Electron microscopy graphs showing lipid droplets, irregular karyotype, and heterochromatin side set of Leydig cells in the DIO group (b), DEHP group (c), DIO + DEHP low group (d), DIO + DEHP middle group, (e) and DIO + DEHP high group (f) compared to the control group (a).

Electron microscopy was performed on the mouse testes in week 12. In the control group (Figure 4(a)), abundant organelles were found in the Leydig cells. We also found smooth and rough endoplasmic reticulum, with only minimal lysosomes and lipid droplets. The chromatin had a light color, the Leydig cells had normal morphology. In the DIO group (Figure 4(b)), DEHP group (Figure 4(c)), DIO + DEHP low group (Figure 4(d)), and the DIO + DEHP middle group, the cytoplasm and organelles were reduced. The mitochondria were swollen and deformed with an increased number of lipid droplets. An irregular karyotype and heterochromatin side set was identified in the Leydig cells from the DIO group (Figure 4(b)), DEHP group (Figure 4(c)), DIO + DEHP low group (Figure 4(d)), and the DIO + DEHP middle group (Figure 4(e)). In the DIO + DEHP high group (Figure 4(f)), the Leydig cells showed vacuolization of the nucleus and cytoplasm; the mitochondria were swollen and deformed, and the number of organelles was reduced.

### 3.5. Serum Sex Hormone and Leptin Levels in the 6 Experimental Groups of Mice

As shown in Table 2, DEHP, DIO, DIO + DEHP low, DIO + DEHP middle, and DIO + DEHP high mice exhibited decreased fasting levels of testosterone at 12 weeks (*P* < 0.05). Furthermore, the testosterone level of DIO + DEHP high mice was significantly lower than either DEHP or DIO mice (*P* < 0.05).

DEHP, DIO + DEHP low, DIO + DEHP middle, and DIO + DEHP high mice exhibited increased fasting levels of estradiol at 12 weeks (*P* < 0.05; Table 2). The levels of estradiol in DEHP, DIO + DEHP low, DIO + DEHP middle, and DIO + DEHP high mice were significantly higher compared to the DIO mice (*P* < 0.01; Table 2).

DEHP, DIO, DIO + DEHP low, DIO + DEHP middle, and DIO + DEHP high mice exhibited increased fasting levels of leptin at 12 weeks (*P* < 0.05; Table 2). The levels of leptin in DIO + DEHP middle and DIO + DEHP high mice were significantly higher compared to the DEHP, DIO, and DIO + DEHP low mice (*P* < 0.01; Table 2).

### 3.6. MDA, T-AOC, SOD, GSH, H_2_O_2_, CAT, and GSH-PX Levels of Testicular Tissue

The effect of obesity and DEHP on biomarkers of oxidative stress is shown in Table 3.

At 12 weeks, obesity and DEHP had caused an increase in the levels of MDA in testis tissue to 130%, 127%, 152%, 155%, and 164% of the control in the DEHP, DIO, DIO + DEHP low, DIO + DEHP middle, and DIO + DEHP high mice, respectively (*P* < 0.01). Furthermore, the levels of MDA in DIO + DEHP low, DIO + DEHP middle, and DIO + DEHP high mice were significantly higher compared to the DEHP and DIO mice (*P* < 0.05; Table 3).

At 12 weeks, the obesity and DEHP had reduced T-AOC levels to 80% and 60% of the controls in the DIO + DEHP middle and DIO + DEHP high mice, respectively (*P* < 0.05). The levels of T-AOC in DIO + DEHP low, DIO + DEHP middle, and DIO + DEHP high mice were significantly lower compared to the DEHP mice (*P* < 0.05). Levels of T-AOC in DIO + DEHP middle and DIO + DEHP high mice were significantly lower compared to the DIO mice (*P* < 0.05). We also found that the levels of T-AOC in DIO + DEHP high mice were significantly lower compared to the DIO + DEHP low mice (*P* < 0.05; Table 3).

At 12 weeks, the obesity and DEHP had reduced SOD levels to 46%, 68.9%, 54%, 45.6%, and 34.7% of the control in the DEHP, DIO, DIO + DEHP low, DIO + DEHP middle, and DIO + DEHP high mice, respectively (*P* < 0.01). Furthermore, the levels of SOD in DIO + DEHP low, DIO + DEHP middle, and DIO + DEHP high mice were significantly lower compared to the DIO mice (*P* < 0.05). We also found that the levels of SOD in DIO + DEHP high mice were significantly lower compared to the DIO + DEHP low mice (*P* < 0.05; Table 3).

At 12 weeks, obesity and DEHP had reduced GSH levels to 87%, 80%, 64%, 41%, and 33% of the controls in the DEHP, DIO, DIO + DEHP low, DIO + DEHP middle, and DIO + DEHP high mice, respectively (*P* < 0.05). Levels of GSH in DIO + DEHP low, DIO + DEHP middle, and DIO + DEHP high mice were significantly lower compared to the DEHP and DIO mice (*P* < 0.01). We also found that the levels of GSH in DIO + DEHP middle and DIO + DEHP high mice were significantly lower compared to the DIO + DEHP low mice (*P* < 0.01; Table 3).

At 12 weeks, obesity and DEHP had increased H_2_O_2_ levels by 2.53-fold, 2.65-fold, 3.13-fold, and 3.68-fold relative to the controls in the DIO, DIO + DEHP low, DIO + DEHP middle, and DIO + DEHP high mice, respectively (*P* < 0.01). The levels of H_2_O_2_ in DIO + DEHP low, DIO + DEHP middle, and DIO + DEHP high mice were significantly higher compared to the DEHP mice (*P* < 0.05). Furthermore, levels of H_2_O_2_ in DIO + DEHP middle and DIO + DEHP high mice were significantly higher compared to the DIO mice (*P* < 0.05). We also found that levels of H_2_O_2_ in DIO + DEHP middle and DIO + DEHP high mice were significantly higher compared to DIO + DEHP low mice (*P* < 0.05). Furthermore, the high-fat diet and DEHP had increased H_2_O_2_ levels by 1.18-fold in the DIO + DEHP middle and the DIO + DEHP high mice (*P* < 0.05; Table 3).

At 12 weeks, obesity and DEHP had reduced CAT levels to 88%, 90%, 89%, 83%, and 71% of the controls in the DEHP, DIO, DIO + DEHP low, DIO + DEHP middle, and DIO + DEHP high mice, respectively (*P* < 0.01). CAT levels in DIO + DEHP high mice were significantly lower compared to DEHP mice (*P* < 0.05). Furthermore, the levels of CAT in the DIO + DEHP middle and DIO + DEHP high mice were significantly lower compared to DIO mice (*P* < 0.05). We also found that the levels of CAT in DIO + DEHP middle and DIO + DEHP high mice were significantly lower compared to DIO + DEHP low mice (*P* < 0.05; Table 3).

At 12 weeks, the high-fat diet and DEHP had reduced GSH-PX levels to 73%, 71%, 53%, and 47% of the controls in the DIO, DIO + DEHP low, DIO + DEHP middle, and DIO + DEHP high mice, respectively (*P* < 0.01). The levels of GSH-PX in DIO + DEHP low, DIO + DEHP middle, and DIO + DEHP high mice were significantly lower compared to DEHP mice (*P* < 0.05. Furthermore, the levels of GSH-PX in DIO + DEHP middle and DIO + DEHP high mice were significantly lower compared to DIO mice (*P* < 0.05). We also found that the levels of GSH-PX in DIO + DEHP middle and DIO + DEHP high mice were significantly lower compared to the DIO + DEHP low mice (*P* < 0.05; Table 3).

### 3.7. Expression of Nrf2 Protein in the 6 Experimental Groups of Mice

**Figure 5:**
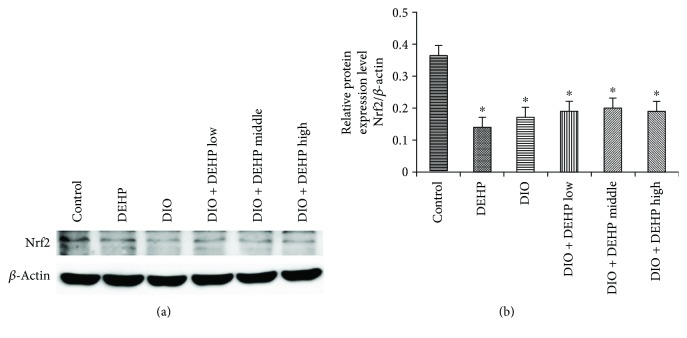
Expression of Nrf2 protein in the 6 experimental groups of mice. The upper bands (a) depict representative findings in the control, DIO, DEHP, DIO + DEHP, DIO + DEHP middle, and DIO + DEHP high groups. The lower bar graphs (b) show the results of the semiquantitative measurement of Nrf2. Each bar represents mean ± SE. *n* = 4. ∗ indicates a significant difference from the control group, *P* < 0.05.

Levels of Nrf2 protein were detected by Western blotting. Results indicated that Nrf2 expression was inhibited in the DEHP, DIO, DIO + DEHP low, DIO + DEHP middle, and DIO + DEHP high groups compared to the control group (Figure 5). Obesity and DEHP significantly reduced levels of Nrf2 protein to 39%, 48%, 53%, 55%, and 52% of the controls in the DEHP, DIO, DIO + DEHP low, DIO + DEHP middle, and DIO + DEHP high group (*P* < 0.05).

### 3.8. Expression of Keap1 Protein in the 6 Experimental Groups of Mice

**Figure 6:**
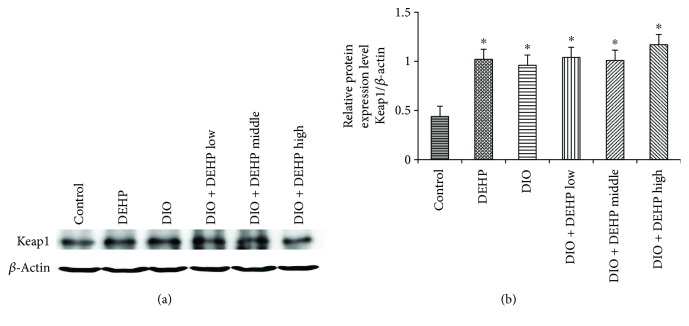
Expression of Keap1 Protein in the 6 experimental groups of mice. The upper bands (a) depict representative findings in the control, DIO, DEHP, DIO + DEHP, DIO + DEHP middle, and DIO + DEHP high groups. The lower bar graphs (b) show the results of the semiquantitative measurement of Keap1. Each bar represents mean ± SE. *n* = 4. ∗ indicates a significant difference from the control group, *P* < 0.05.

Levels of Keap1 protein were detected by Western blotting. Results indicated that Keap1 expression increased in the DEHP, DIO, DIO + DEHP low, DIO + DEHP middle, and DIO + DEHP high groups compared to the control group (Figure 6). Obesity and DEHP significantly increased the levels of Keap1 protein by 130%, 117%, 135%, 129%, and 167% in the DEHP, DIO, DIO + DEHP low, DIO + DEHP middle, and DIO + DEHP high groups, respectively, compared to the controls (*P* < 0.05).

## 4. Discussion

In this study, the weight of DEHP mice was higher than the control mice; however, there was no significant difference in weight between the DEHP and control group prior to exposure to DEHP. We found that DEHP, as an estrogenic endocrine disruptor, may increase the weight of mice. Kim et al. found that DEHP exposure may affect body mass change in early life through changes of obesity-related markers [17]. In another study, Lv et al. suggested that chronic DEHP exposure could induce obesity by interrupting energy homeostasis [18]. After joint exposure to a high-fat diet and DEHP, the weight of DIO + DEHP low, DIO + DEHP middle, and DIO + DEHP high mice were higher than the control mice, although this difference was only statistically significant in the DIO + DEHP middle mice. However, the weight of DIO + DEHP low, DIO + DEHP middle, and DIO + DEHP high DIO mice was lower than the DIO mice. Consequently, we did not identify a joint effect of DEHP and obesity upon body weight in mice. There are three possible reasons for this: (1) there was a different mechanism of body weight gain between the high-fat diet and DEHP; (2) the exposure time was not long enough; and (3) DEHP exerted a toxicity effect. The joint effect of a high-fat diet and DEHP upon body weight should therefore be investigated more thoroughly.

Twelve weeks after joint exposure to a high-fat diet and DEHP, we found the following effects: (1) a reduction in the relative epididymis coefficient; (2) a decline in sperm motility; and (3) pathological damage to the Leydig cells (as shown by both light microscopy and transmission electron microscopy). Consequently, male obesity and DEHP may concomitantly cause hypogonadism.

Testosterone is the most important sex hormone in males and plays a critical role in testis development, spermatogenesis, and the maintenance of normal masculinization. Other studies have found that lower plasma testosterone levels played an important role in male hypogonadism caused by obesity [7, 8]. In this study, we identified significantly lower serum testosterone levels in DEHP, DIO, DIO + DEHP low, DIO + DEHP middle, and DIO + DEHP high mice when compared to the control group. Furthermore, the testosterone level of DIO + DEHP high mice was significantly lower than DEHP and DIO mice. Consequently, there was a joint effect leading to reduced testosterone levels in DIO + DEHP high mice.

To investigate the mechanisms underlying the joint effect of DEHP and obesity on low testosterone levels, we determined the levels of leptin and oxidative stress in the testicular tissue. Leptin is expressed predominantly in adipose tissue and can reduce appetite and increase energy expenditure [19]. Leptin also plays an important role in male reproduction [20, 21]. Leptin receptors are also distributed in the testicular tissue [22] which suggests that leptin has a direct effect upon the testis. Previous work has found that leptin levels are inversely correlated with testosterone level in both boys and adult males [23, 24]. In the present study, DEHP, DIO, DIO + DEHP low, DIO + DEHP middle, and DIO + DEHP high mice exhibited increased fasting levels of leptin at 12 weeks. The serum concentration of leptin was inversely correlated with testosterone at 12 weeks. Consequently, in line with our previous findings [9], our present data indicate that leptin levels in obese mice were higher than in normal mice. Isidori et al. also found a clear relationship between high leptin levels and low testosterone levels in obese males [25]. High levels of leptin can inhibit testosterone levels in obese males [25].

DEHP is known to exert weak estrogenic properties. Furthermore, DEHP acts as an endocrine disruptor owing to its ability to compete with endogenous steroid hormones binding to receptors [26]. There is sufficient evidence in rodents that phthalate exposure causes developmental and reproductive toxicities; DEHP may cause dysmorphic disorders of the genital tract in infant males [27]. Rats exposed to DBP/BBP/MBP (toxic metabolite products of DEHP) during the perinatal period are known to induce reproductive disorders, such as low sperm counts [28]. Lv et al. suggested that chronic DEHP exposure could induce obesity and increase leptin levels [18] while Sena et al. found that tributyltin chloride (a type of environmental estrogen) can increase leptin level in female rats [29]. In the present study, compared to the control mice, the leptin levels of DEHP mice were higher, and the testosterone levels were lower. DEHP may also increase the levels of leptin in mice, in a manner similar to the fact that high leptin levels can inhibit testosterone levels in obese males. High leptin levels can inhibit testosterone levels during exposure to DEHP. In order to identify if there was a joint effect on leptin level when mice were jointly exposed to obesity and DEHP, we designed an experiment featuring 3 groups for 3 levels of DEHP exposure. We found that the levels of leptin in DIO + DEHP middle and DIO + DEHP high mice were significantly higher compared to DEHP, DIO, and DIO + DEHP low mice. Thus, obesity and DEHP had a joint effect on leptin level. That is to say that high leptin levels may be one of the main mechanisms underlying the low testosterone level caused by the concomitant exposure of mice to obesity and DEHP.

With regards to the mechanism of high leptin levels and low testosterone observed in obese mice in this study, Smith et al. found that high leptin levels were related to low kisspeptin levels [30]. High leptin may reduce testosterone level by downregulating the expression of kisspeptin [31]. Yuan et al. considered that a reduced p-STAT3 protein level in testicular tissue was related to leptin resistance and sex hormone dysregulation [32]. Yi et al. further found that obesity can inhibit testosterone biosynthesis by disrupting the testicular leptin transduction pathway (LEP–JAK2–STAT3 signal pathway) in the testis [33]. Therefore, the role of high leptin inhibition not only occurs at the hypothalamic-pituitary level but also at the gonadal level. The mechanisms underlying the joint effect of obesity and DEHP on leptin level should be studied further.

Oxidative stress in testicular tissue is another important factor to consider. Oxidative stress results from the production of oxygen radicals in excess of the antioxidant capacity of the stressed tissue. Increasing testicular oxidative stress may lead to subsequent hypospermatogenesis [34].

Testicular oxidative stress may also be associated with reduced testosterone levels in obese males [9, 35]. To investigate the mechanisms of low testosterone levels induced by the joint exposure to obesity and DEHP, we determined some markers of oxidative stress: MDA, T-AOC, SOD, GSH, H_2_O_2_, CAT, and GSH-PX expression.

At 12 weeks, MDA and H_2_O_2_ levels were higher in the DEHP, DIO, DIO + DEHP low, DIO + DEHP middle, and DIO + DEHP high mice than in the control group. Furthermore, the levels of MDA and H_2_O_2_ in DIO + DEHP low, DIO + DEHP middle, and DIO + DEHP high mice were significantly higher compared to DEHP and DIO mice. There is evidence that H_2_O_2_, besides acting as independent signaling molecules, may also interrelate to form an oxidative death cycle [34, 36]. Obesity and DEHP jointly caused oxidative damage within the testicular tissue. These results suggest that obesity and DEHP induced excessive oxidative stress and may affect the histological structure and function of the testicular tissue.

The levels of several antioxidant enzymes (SOD, GSH, CAT, and GSH-PX) were found to be reduced in DEHP, DIO, DIO + DEHP low, DIO + DEHP middle, and DIO + DEHP high mice when compared to the control group at 12 weeks. Similar to our prior study, Erdemir et al. also found that SOD and GSH-PX were reduced in male rat offspring when the mother was obese [37]. Other researches have shown that exposure to endocrine disruptors which diminish the intratesticular concentration of testosterone may also inhibit the testicular expression of antioxidant enzymes such as GPx, SOD, and catalase [38, 39]. We also found that the levels of GSH, CAT, and GSH-PX in DIO + DEHP high mice were significantly lower compared to DEHP and DIO mice. Obesity and DEHP jointly reduced the level of antioxidant enzymes in the male testis tissue.

This study tries to identify the mechanism underlying the effect of oxidative stress caused by the joint effects of obesity and DEHP. NFE2-related factor 2 (Nrf2) is a central regulator of antioxidant and detoxification gene expression in response to electrophilic or oxidative stress [40]. Under homeostatic conditions, Nrf2 is repressed via cytoplasmic tethering and ubiquitination, mediated by the redox-sensitive Kelch-like ECH-associated protein 1 (Keap1) [41–43] and is constitutively degraded via the ubiquitin-proteasome pathway in the cytoplasm [43]. In this study, Keap1 expression increased while Nrf2 expression decreased along with the activities of other enzymatic antioxidants. Thus, oxidative stress caused by obesity and DEHP in the testes may be improved by inhibiting the Nrf2 antioxidant pathway. Thus, high MDA and H_2_O_2_ levels, and low GSH, CAT, and GSH-PX levels, may contribute to the low levels of testosterone induced by obesity and DEHP in testicular tissue. The low expression of Nrf2 and high expression of Keap1 may have contributed to the low expression of GSH, CAT, and GSH-PX.

In conclusion, the joint exposure of mice to obesity and DEHP caused pathological damage to the Leydig cells, increased serum leptin levels, and caused reductions in sperm count, motility, relative epididymis weight, and testosterone level. The activity of GSH, CAT, and GSH-PX enzymes was also reduced, as was the expression of Nrf2 . However Keap1 expression increased. We conclude that high levels of leptin and oxidative stress in testicular tissue may provide some evidence to clarify the mechanisms of male SH in obesity and DEHP.

## Figures and Tables

**Table 1:** The reproductive organs weight, sperm motility, count, and retroperitoneal, and epididymal fat weight in 12 weeks ($\bar{x}$ ± SD).

Group	*n*	Relative Tes. weight (g/100 g)	Relative epididymis weight (g/100 g)	Relative Sem. weight (g/100 g)	Relative liver weight (g/100 g)	Relative kidney weight (g/100 g)	Relative Epi fat (g/100 g)	Relative Ret fat (g/100 g)	Sperm motility (%)	Sperm count (×10^6^/ml)
Control	10	0.85 ± 0.06	0.29 ± 0.02	0.98 ± 0.11	4.48 ± 0.30	1.36 ± 0.10	1.25 ± 0.37	0.26 ± 0.09	31.03 ± 3.08	4.75 ± 0.50
DEHP	10	0.71 ± 0.06^b^	0.27 ± 0.01^b^	0.96 ± 0.14	3.97 ± 0.24^b^	1.24 ± 0.04^b^	2.47 ± 0.61^b^	0.69 ± 0.16^b^	23.47 ± 2.24^b^	4.05 ± 0.44^b^
DIO	10	0.74 ± 0.07^b^	0.26 ± 0.01^b^	0.94 ± 0.11	3.83 ± 0.16^b^	1.24 ± 0.07^b^	3.04 ± 0.82^b^	0.92 ± 0.37^b^	23.33 ± 3.94^b^	3.89 ± 0.41^b^
DIO + DEHP low	10	0.69 ± 0.08^b^	0.26 ± 0.01^b^	0.95 ± 0.09	3.73 ± 0.20^bc^	1.20 ± 0.07^b^	2.35 ± 0.39^bf^	0.68 ± 0.16^bef^	20.49 ± 2.03^bce^	3.60 ± 0.44^bc^
DIO + DEHP middle	10	0.69 ± 0.14^b^	0.25 ± 0.02^bc^	0.95 ± 0.18	3.52 ± 0.20^bdf^	1.17 ± 0.03^bce^	1.84 ± 0.50^adf^	0.45 ± 0.15^af^	19.19 ± 1.17^bdf^	3.51 ± 0.42^bc^
DIO + DEHP high	10	0.69 ± 0.05^b^	0.23 ± 0.02^bdfhj^	0.89 ± 0.16	3.32 ± 0.23^bdf^	1.14 ± 0.05^bdf^	1.70 ± 0.25^df^	0.42 ± 0.13^df^	17.15 ± 1.78^bdf^	3.42 ± 0.50^bde^

Data are mean ± SD. ^b^*P* < 0.01 denotes statistical significance compared with the control group; ^c^*P* < 0.05 and ^d^*P* < 0.01 denote statistical significance compared with the DEHP group; ^e^*P* < 0.05 and ^f^*P* < 0.01 denote statistical significance compared with the DIO group; ^h^*P* < 0.01 denotes statistical significance compared with the DIO + DEHP low group; ^j^*P* < 0.01 denotes statistical significance compared with the DIO + DEHP middle group. Testis: Tes; seminal vesicles: Sem; retroperitoneal: Ret. epididymal: Epi. Relative Tes. weight = testis weight/body weight × 100. Relative epididymis weight = epididymis weight/body weight × 100. Relative Sem. weight = seminal vesicle weight/body weight × 100. Sperm motility = total motile sperm/all count sperm × 100. Relative liver weight = liver weight/body weight × 100. Relative kidney weight = kidney weight/body weight × 100. Relative Ret fat weigh = retroperitoneal fat weight/body weight × 100. Relative Epi fat weight = epididymal fat weight/body weight × 100.

**Table 2:** Testosterone, estradiol, and leptin level in the 6 experimental groups ($\bar{x}$ ± SE).

Group	*n*	Testosterone (ng/ml)	Estradiol (ng/ml)	Leptin (ng/ml)
Control	10	4.38 ± 0.15	29.69 ± 2.38	1.71 ± 0.11
DEHP	10	2.99 ± 0.23^b^	80.36 ± 9.69^b^	4.01 ± 0.72^a^
DIO	10	3.02 ± 0.21^b^	47.14 ± 7.01^d^	4.07 ± 0.81^a^
DIO + DEHP low	10	2.35 ± 0.42^b^	90.42 ± 6.35^bf^	6.01 ± 0.76^b^
DIO + DEHP middle	10	2.36 ± 0.30^b^	94.04 ± 6.91^bf^	9.10 ± 0.95^bdfh^
DIO + DEHP high	10	2.18 ± 0.23^bce^	86.49 ± 10.00^bf^	10.04 ± 0.68^bdfh^

Data are mean ± SE. ^a^*P* < 0.05 and ^b^*P* < 0.01 denote statistical significance compared with control group; ^c^*P* < 0.05 and ^d^*P* < 0.01 denote statistical significance compared with the DEHP group; ^e^*P* < 0.05 and ^f^*P* < 0.01 denote statistical significance compared with the DIO group; ^h^*P* < 0.01 denotes statistical significance compared with the DIO + DEHP low group.

**Table 3:** MDA, T-AOC, SOD, GSH, H_2_O_2_, CAT, and GSH-PX levels of testis tissue of the 6 experimental groups in 12 weeks ($\bar{x}$±SD).

Group	*n*	MDA (nmol/mg prot)	T-AOC (U/mg prot)	SOD (U/mg prot)	GSH (mgGSH/g prot)	H_2_O_2_ (mmol/g prot)	CAT (U/mg prot)	GSH-PX (U/mg prot)
Control	10	3.3 ± 0.5	0.5 ± 0.2	196.7 ± 37.7	114.8 ± 15.6	3.1 ± 0.58	16.0 ± 0.9	55.7 ± 6.8
DEHP	10	4.3 ± 0.5^b^	0.5 ± 0.2	90.5 ± 28.4^b^	99.5 ± 13.5^a^	2.8 ± 0.61	14.1 ± 1.0^b^	49.8 ± 10.9
DIO	10	4.2 ± 0.8^b^	0.5 ± 0.1	135.6 ± 19.4^b^	91.8 ± 16.3^b^	7.6 ± 1.7^b^	14.4 ± 1.8^b^	40.4 ± 7.1^b^
DIO + DEHP low	10	5.0 ± 0.7^bce^	0.4 ± 0.1^c^	106.2 ± 25.6^be^	73.7 ± 17.6^bdf^	8.2 ± 1.5^bd^	14.3 ± 1.1^b^	39.3 ± 5.6^bc^
DIO + DEHP middle	10	5.1 ± 0.7^bcf^	0.4 ± 0.1^ade^	89.6 ± 35.2^bf^	47.6 ± 11.1^bdfh^	9.7 ± 2.36^bdfg^	13.2 ± 0.9^beh^	29.5 ± 3.1^bdeg^
DIO + DEHP high	10	5.4 ± 0.7^bdf^	0.3 ± 0.1^bdfg^	68.4 ± 10.9^bfh^	38.2 ± 11.8^bdfh^	11.4 ± 2.2^bdfhi^	11.4 ± 1.0^bdfh^	26.3 ± 6.2^bdfh^

Data are mean ± SD. ^a^*P* < 0.05 and ^b^*P* < 0.01 denote statistical significance compared with the control group; ^c^*P* < 0.05 and ^d^*P* < 0.01 denote statistical significance compared with the DEHP group; ^e^*P* < 0.05 and ^f^*P* < 0.01 denote statistical significance compared with the DIO group; ^g^*P* < 0.05 and ^h^*P* < 0.01 denote statistical significance compared with the DIO + DEHP low group; ^i^*P* < 0.05 denotes statistical significance compared with the DIO + DEHP middle group.
